# Visible Light-Driven Alkyne-Grafted Ethylene-Bridged Azobenzene Chromophores for Photothermal Utilization

**DOI:** 10.3390/molecules27103296

**Published:** 2022-05-20

**Authors:** Wenyu Fang, Yiyu Feng, Jian Gao, Hui Wang, Jing Ge, Qingbin Yang, Wei Feng

**Affiliations:** School of Materials Science and Engineering, Tianjin University, Tianjin 300350, China; fangwenyu@tju.edu.cn (W.F.); gj1996@tju.edu.cn (J.G.); huiwang1928@tju.edu.cn (H.W.); gejing_0520@tju.edu.cn (J.G.); yqb514@tju.edu.cn (Q.Y.)

**Keywords:** azobenzene, photoresponse, visible-light driven, solar energy storage, photothermal utilization

## Abstract

Molecular photoswitches are considered to be important candidates in the field of solar energy storage due to their sensitive and reversible bidirectional optical response. Nevertheless, it is still a daunting challenge to design a molecular photoswitch to improve the low solar spectrum utilization and quantum yields while achieving charging and discharging of heat without solvent assistance. Herein, a series of visible-light-driven ethylene-bridged azobenzene (b-Azo) chromophores with different alkyne substituents which can undergo isomerization reactions promoted in both directions by visible light are reported. Their visible light responsiveness improves their solar spectrum utilization while also having high quantum yields. In addition, as the compounds are liquids, there is no need to dissolve the compounds in order to exploit this switching. The photoisomerization of b-Azo can be adjusted by alkyne-related substituents, and hexyne-substituted b-Azo is able to store and release photothermal energy with a high density of 106.1 J·g^−1^, and can achieve a temperature increase of 1.8 °C at a low temperature of −1 °C.

## 1. Introduction

Photochemical conversion reactions are generally considered to be among the most promising methods for converting light energy into latent heat [1]. There are several materials under research that can be applied to photothermal conversion, including spiro-pyran, azobenzene, and norbornadiene, and they have important applications in photochemistry, sensing, energy, biomedicine, and other fields [2,3,4,5,6,7]. Among them, azobenzene chromophores (Azo) [8,9] have received considerable attention due to their excellent reversible *trans*(*E*)-*cis*(*Z*) photoisomerizations [10,11]. *E*-Azo compounds usually absorb photons at characteristic wavelengths, which cause transformations into their metastable *Z*-isomers. These transformations are accompanied by heat storage. These isomers then release heat when stimulated by light at another wavelength, by returning to the stable *E*-state; this is a closed photothermal energy cycle [12]. However, *E*-Azo can often only absorb UV light at wavelengths in the range of 300–350 nm, as limited by their molecular energy levels. However, the UV energy only accounts for 5–7% of the total solar energy, and it has non-negligible harmful effects on the human body and materials, so it is meaningful to research and develop visible light-driven Azo [13]. Furthermore, strong molecular π-stacking hinders the isomerization of solid *E*-Azo without solvent assistance [14].

Bridged Azo compound (b-Azo) is regarded as an important candidate for visible light-responsive energy utilization, as its absorption peak is red-shifted into the visible region [15]. Different from ordinary azobenzene, b-Azo is stable in the *Z* configuration. Its *Z* isomer can be switched to *E* isomer with an efficiency >90% by using blue light at λ ≈ 370–420 nm, and it also has high quantum conversion efficiencies [16,17,18,19]. However, the switching efficiencies of functionalized b-Azo compounds are also generally low, similar to azobenzene. This has been reported for an amine-substituted b-Azo [20,21], the electronic coupling of which leads to overlapping absorption bands. The degree of isomerization (the proportion of the metastable *E*-isomers accounted for) of this compound is only 30% after illumination at its excitation wavelength [16,17,18,19,22]. Herges et al. [22] proposed that the electronic decoupling of the azobenzene unit from oxygen- and nitrogen-containing functional groups could be achieved by the use of CH_2_ groups, to avoid the overlap of absorption bands. Therefore, alkyne chains with weak electronic coupling are used as substituents in this paper. This not only avoids the overlap of absorption bands and improves the degree of isomerization but also disrupts the crystallinity and reduces molecular stacking effects. Hence, these compounds achieve heat charging at low temperature and without the presence of solvent [23]. Therefore, grafting alkyne chains onto a b-Azo is hypothesized to be an effective strategy to regulate its isomerization process and its photothermal output performance.

Here, we report a series of novel b-Azo compounds. These azobenzene systems show fast and efficient visible light responses and have good cycling performances. They can also achieve fast and controlled heat charge and release cycles, without solvent assistance and over a wide temperature range. The degree of isomerization and energy density can be regulated by changing the substituents to further modulate the photothermal output process.

## 2. Results and Discussion

### 2.1. Basic Properties

**Figure 1:**
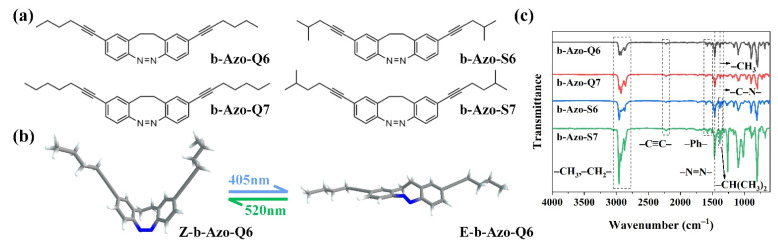
(**a**) Chemical structures of the four b-Azo chromophores; (**b**) Z-E isomers of b-Azo-Q6; (**c**) FTIR spectra of four b-Azo chromophores.

After improvement according to the literature method [17,24], we designed and synthesized two b-Azo molecules (b-Azo-Q6, b-Azo-Q7) by an intramolecular Baeyer–Mills reaction and a Sonogashira coupling reaction. Two b-Azo molecules (b-Azo-S6, b-Azo-S7) with isopropyl groups were also designed and synthesized to investigate the effect of the terminal groups on the properties of the b-Azo [25]. The structural formulae of the four compounds are shown in Figure 1a, and all of these compounds are viscous liquids at room temperature (Figure S18, Supplementary Materials). As an example, Figure 1b shows b-Azo-Q6 in both of its forms; similarly, all four b-Azo chromophores can adopt the boat-*cis* conformation and the twisted-*trans* conformation [15]. The successful synthesis of the four compounds was determined by Fourier transform infrared spectroscopy (FTIR), high-resolution mass spectra (HRMS), ^1^H NMR and ^13^C NMR (Figure 1c and Figures S6–S17, Supplementary Materials).

### 2.2. Photoisomerization

**Figure 2:**
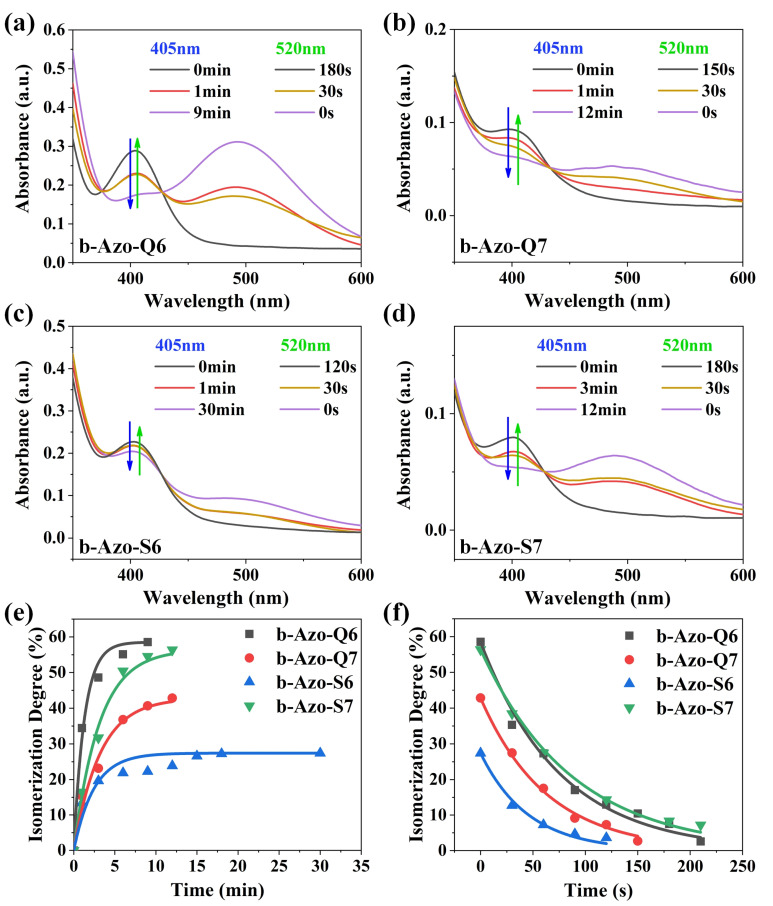
(**a**–**d**) UV–vis absorption spectra of the four b-Azo chromophores in the liquid state. Samples were taken in the neat state and dissolved in EtOAc, and the spectra had been normalized with respect to the isosbestic point at 427.5 nm. (**e**) Degrees of isomerization of the four b-Azo chromophores during charging versus time under 405 nm of blue light irradiation. (**f**) Degrees of isomerization of the four b-Azo chromophores during reversion versus time under 520 nm of green light irradiation.

Unlike azobenzene, the *Z*-*E* isomerization of b-Azo can be completely controlled with visible light, using blue light for heat charging and green light to reverse it, releasing the stored energy. This avoids UV damage to the material. The charging performance of the compounds under blue light irradiation (405 nm, 24.01 mW·cm^−2^) and their exothermic performance under green light irradiation (520 nm, 5.06 mW·cm^−2^) was investigated by UV–vis absorption spectroscopy, as shown in Figure 2a–d. Because they are liquids at room temperature and have good light transmittance, they can be charged without solvent assistance [26], which is a notable advantage over azobenzene. In the testing process, a light source is used to irradiate liquid samples directly, and then a small amount of samples are scraped at a specific time to dissolve in a solvent for testing.

Additionally, the results show that the four compounds had excellent visible light responsiveness: with blue light irradiation, the *Z*-isomer gradually transformed into the *E*-isomer, and the peak at 400 nm decreased, while the peak at 500 nm increased; with green light irradiation, the *E*-isomer gradually returned and the absorption peak progressively changed back to the initial state.

Nuclear magnetic resonance was used to study the isomerization behavior of the b-Azo chromophores under blue light irradiation, the chemical shifts of which will change due to the different configurations of the *Z* and *E* isomers [27]. The degree of isomerization can be calculated from the peak integration (Figures S6–S17, Supplementary Materials). It can be calculated that the maximum degrees of isomerization of b-Azo-Q6, b-Azo-Q7, b-Azo-S6 and b-Azo-S7 are 58.5%, 42.8%, 27.4% and 56.4%, respectively. It should be noted that the configurations of these compounds are inherently distorted, and coupled with the effect of rigid alkyne substitution, small changes in the length and volume of the molecular chain have a significant impact on intermolecular forces, steric hindrance, planarity and so on [17,28]. It can be seen that b-Azo-Q6 has the highest degree of isomerization, a reason may be that it has the shortest chain length and thus the smallest resistance to molecular motion. In contrast, b-Azo-S6 has a low degree of isomerization, which is perhaps due to the larger resistance caused by the isopropyl group. Another reason is the poor planarity of the *E* isomer which weakens the intermolecular forces and makes the isomer less stable. In the case of b-Azo-S7, the longer molecular chain improves the planarity of the *E* isomer to a certain extent, thus increasing the stability of the isomer [28,29].

The degree of isomerization versus time curves for the two processes are shown in Figure 2e,f. During the charging process, b-Azo-Q6 has the highest degree of isomerization and the shortest charging time; this can still be attributed to its smaller steric hindrance and the high planarity of the *E* isomer. It therefore has the highest charging efficiency and is the best candidate in this series for use as a solar thermal fuel. Except b-AZo-S6, the other three compounds can be charged within 10 min, which is considerably faster than for azobenzene. The rate constants that their exothermic rates are comparable; the discharge times depend only on the maximum degree of isomerization. In addition, the graphs show that all of the compounds have a fast and efficient heat release and the exothermic discharge processes for each are complete within 3 min [25,28,29].

### 2.3. Thermal Performance and Visual Photo-Driven Heat Release

**Figure 3:**
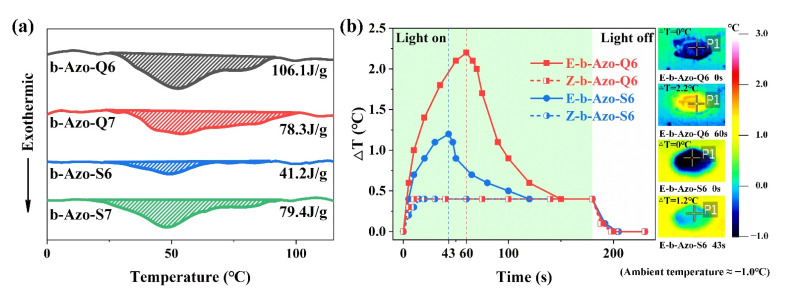
(**a**) DSC exothermic curves for the four charged b-Azo chromophores between 0 and 110 °C at a heating rate of 2 °C/min; (**b**) ΔT versus time graphs for the exothermic discharge processes of b-Azo-Q6 and b-Azo-S6, and infrared thermal images at several time points. P1 is the highest temperature inside the sample. The ambient temperature is −1 °C.

The exothermic processes of the four b-Azo chromophores were monitored by differential scanning calorimetry after being charged by blue light irradiation, as shown in Figure 3a. From these experiments, the enthalpies of isomerization (ΔH_iso_) of the four b-Azo chromophores were calculated. The isomerization enthalpies are expected to mainly depend on the degree of isomerization. Among the compounds, b-Azo-Q6 has the highest degree of isomerization and the highest energy density, which can reach a value of 106.1 J·g^−1^.

It was found that the exothermic temperatures of the b-Azo compounds are all much lower than those of azobenzene [30], so we investigated their suitability for use in low-temperature environments. b-Azo-Q6 was coated onto a glass plate, charged with blue light for 15 min, and was then placed onto a cold table (below 0 °C). When the temperature in the center of the sheet stabilized at approximately −1 °C, the sample was irradiated with green light to stimulate the exothermic and the temperature was tracked using a high-resolution infrared thermal imaging camera. The resultant graph of temperature difference (ΔT) versus time is shown in Figure 3b for *E*-b-Azo-Q6 [31]. In addition, the uncharged *Z*-b-Azo-Q6 was monitored under the same green light irradiation conditions to exclude the heating effect of the light source and the photothermal effect of the material [31,32]. The exothermic process of b-Azo-S6 was monitored by the same method for comparison, and the results for *E*-b-Azo-S6 and *Z*-b-Azo-S6 are shown in Figure 3b.

It can be seen in the figure that the temperature of all four samples rose rapidly after the start of irradiation. The ΔT of *E*-b-Azo-S6 reached a maximum of 1.2 °C after 43 s, whereas the ΔT of *E*-b-Azo-Q6 reached a maximum of 2.2 °C after 60 s. This proves that b-Azo-S6 discharges faster than b-Azo-Q6, but the ΔT of b-Azo-Q6 is higher because of its higher energy density. Both materials can complete heat release within 3 min, which is consistent with the response process under green light irradiation. The experiments with the *Z*-isomers demonstrate that the exothermic curves contain part of the heat of the light source and the photothermal effect of the material. The difference in ΔT between the *Z* and *E* isomer curves is the temperature difference due to isomerization (ΔT_iso_). The ΔT_iso_ of b-Azo-Q6 and b-Azo-S6 are 1.8 and 0.8 °C, respectively. This experiment proves that this series of liquid b-Azo compounds can perform fast and effective exothermic processes at low temperatures.

The results show that this series of azobenzene molecules can be driven at low temperatures using visible light to achieve storage of light energy or release of heat while undergoing photoisomerization, thus causing reversible temperature changes. Since this series of molecules does not require the use of ultraviolet light irradiation and avoids harm to humans and materials, it expands the application of photothermal materials in fields such as wearable insulation materials. In addition, this heat release at low temperatures proves its potential as a thermal management material with important application prospects in fields such as deicing, temperature control systems in space stations, warm clothing, and solar blankets [8].

## 3. Materials and Methods

### 3.1. Materials

Unless otherwise noted, the reagents were obtained from commercial sources and used as received. All other chemicals, including 2,2′-ethylenedianiline, were purchased from Bide Pharmatech Ltd., Shanghai, China.

### 3.2. Measurements

The chemical structures of the Azo compounds were characterized using Fourier transform infrared spectroscopy (Tensor 27 spectrometer, Bruker, Billerica, MA, USA) in a KBr disc. ^1^H NMR spectra were collected using a 400 MHz spectrometer (INOVA, Varian, Palo Alto, CA, USA) with tetramethylsilane as an internal standard. ^13^C NMR spectra were collected using a 600 MHz spectrometer (JNM-ECZ600R/S1, JEOL, Akishima, Japan). HRMS were measured using Thermo Scientific Q Exactive Modular Orbitrap mass Spectrometer (Thermo Fisher Scientific, Waltham, MA, USA). The UV–vis absorption spectra of the materials were measured (1 cm path length quartz cuvettes, EA; 330 UV-vis-NIR spectrophotometer, Hitachi, Tokyo, Japan). Differential scanning calorimetry (TA Q20, TA instruments, New Castle, DE, USA) was used to measure the heat flow of the energetically charged *E* isomers of the compounds.

### 3.3. Synthesis of the Intermediate

2,2′-diaminobibenzyl (3 g, 14.1 mmol) was dissolved in DMSO (20 mL) with magnetic stirring at room temperature. To this, a solution of N-iodosuccinimide (6.75 g, 30 mmol) was added in DMSO (45 mL) in three portions over 10 min. The mixture was then stirred at room temperature overnight. Next, dichloromethane (20 mL) and deionized water (150 mL) were added and the solution was stirred for 30 min. The resulting solid was separated by vacuum filtration. Silica gel column chromatography was used to purify the solid residue (2:1 ethyl acetate/petroleum ether) and the solvent was removed under reduced pressure to obtain relatively pure 2,2′-(ethane-1,2-diyl) bis (4-iodoaniline) (5.5 g, 84.4%).

2,2′-(ethane-1,2-diyl) bis (4-iodoaniline) (1 g, 2.2 mmol) was added to a mixture of acetic acid (15 mL) and dichloromethane (45 mL) at room temperature, with stirring. meta-Chloroperoxybenzoic acid (m-CPBA, 85% purity, 7.3 g, 36 mmol) was placed into a brown sample bottle, and glacial acetic acid (60 mL) was added to fully dissolve it. Using a syringe pump, this m-CPBA solution (7.3 mL, 4.4 mmol) was then slowly added to the reaction over 12 h. After the addition was complete, stirring was continued for 2 h. The solvent was removed under reduced pressure, and the crude product was purified by silica gel column chromatography (1:19 ethyl acetate/petroleum ether). The solvent was removed under reduced pressure to obtain (Z)-2,9-diiodo-11,12-dihydrodibenzo [c,g] [1,2] diazocine as a yellow powder (633 mg, 62.5%) [24].

### 3.4. Synthesis of b-Azo-Q6

This step uses the general procedure of the Sonogashira coupling reaction. (Z)-2,9-diiodo-11,12-dihydrodibenzo [c,g] [1,2] diazocine (460 mg, 1 mmol) was added to ultra-dry tetrahydrofuran (10 mL) and triethylamine (556 μL, 4 mmol) in a three-necked flask. To this, a slight excess of 1-hexyne (234 μL, 2.1 mmol), cuprous iodide (9.5 mg, 0.05 mmol) and bis (triphenylphosphine) palladium (II) dichloride (35.1 mg, 0.05 mmol) were added. The mixture was stirred overnight under an inert gas atmosphere. After the reaction was complete, the solvent was removed under reduced pressure and the residue was purified by silica gel column chromatography (7:93 ethyl acetate/petroleum ether). The solvent was removed under reduced pressure to obtain b-Azo-Q6 as a viscous yellow liquid (313 mg, 85.1%).

### 3.5. Synthesis of b-Azo-Q7

This step is the same as the synthesis of b-Azo-Q6, but with 1-heptyne (269 μL, 2.1 mmol) instead of 1-hexyne. b-Azo-Q7 was obtained as a viscous yellow liquid (320 mg, 80.8%).

### 3.6. Synthesis of b-Azo-S6

This step is the same as the synthesis of b-Azo-Q6, but with 4-methyl-1-pentyne (247 μL, 2.1 mmol) instead of 1-hexyne. b-Azo-S6 was obtained as a viscous yellow liquid (300 mg, 81.5%).

### 3.7. Synthesis of b-Azo-S7

This step is the same as the synthesis of b-Azo-Q6, but with 5-methyl-1-hexyne (269 μL, 2.1 mmol) instead of 1-hexyne. b-Azo-S7 was obtained as a viscous yellow liquid (317 mg, 80.1%).

### 3.8. Computational Methods

All calculations were carried out with the Gaussian 16 software. For geometry optimization and frequency calculations, the B3LYP functional and 6-311G(d) basis set were used, and the optimal geometry for each compound was determined [33].

### 3.9. Characterization of Photoisomerization Behavior of b-Azo

The b-Azo compound was irradiated with a 500 W point light source (Shenzhen Height-led Optoelectronics Technology Co., Ltd., Shenzhen, China); 405 and 520 nm lamps were selected. Time intervals were recorded, and a small amount of samples were scraped with toothpicks at specific time points and dissolved in ethyl acetate for testing. The light source was placed 10 cm above the sample. The light intensities were: 405 nm, 24.01 mW/cm^2^; 520 nm, 5.06 mW/cm^2^. The temperature was 25 °C. A spectrophotometric analysis of the charging process was performed using 405 nm blue light irradiation at room temperature. Test until the sample reaches the maximum degree of isomerization. A spectrophotometric analysis of the heat recovery process under ambient conditions was performed by covering a sample of the *E* isomer with tin foil and keeping it in the dark while the measurements were taken. A spectrophotometric analysis of the heat recovery process was also performed under 520 nm green light irradiation at room temperature. All UV–vis absorption spectroscopy images have been normalized [28]. And compared the rates by calculating the first-order kinetic constants (Figure S22, Supplementary Materials) [34].

### 3.10. Cyclic Stability of Isomerization

From an initial UV–vis absorption spectra of the compounds, the intensities of the maximum absorption peaks in the blue and in the green regions were recorded. The compounds were irradiated with blue light for 15 min and the blue and green absorption maxima were recorded. The compounds were then irradiated with green light for 5 min, and the absorption maxima were recorded again. This cycle of blue and green light irradiation was repeated 10 times to demonstrate the cycle stability of the compounds.

### 3.11. Visual Characterization of Infrared Thermal Imaging Camera Temperature Changes

Real-time temperature changes during the sample exotherm were monitored by infrared thermography for visual characterization. A 20 mg sample was weighed and coated onto a quartz sheet, and the sample was irradiated using a blue point light source (405 nm, 24.01 mW/cm^2^) for 15 min. After heat charging, it was placed on a semiconductor-cooled thermostatic cold table set at −3 °C. The sample temperature was allowed to stabilize; at this time, the central temperature of the sample was maintained at approximately −1 °C. The sample was irradiated with a green point light source (520 nm, 5.06 mW/cm^2^) to stimulate the heat discharging process, while the temperature change during the sample exotherm was monitored using an infrared thermal imaging camera.

## 4. Conclusions

In summary, this series of bridged azobenzene derivatives can use different wavelengths of visible light to store and release energy without solvent assistance. This constitutes a controlled and reversible temperature change process, which can be used in applications at low temperatures. We have prepared four azobenzene chromophores that enable the use of visible light by introducing a bridged structure, which avoids the side effects that the use of UV light can have on the material. Long carbon chains are grafted onto these compounds to prevent crystallization and achieve an amorphous state that can be maintained over a wide temperature range. A high degree of isomerization can thus be achieved without solvent assistance, while ensuring high energy densities. These compounds are molecular photoswitches that will be very valuable for research. This study provides a more convenient method to design b-Azo chromophores without destroying their own UV–vis absorption properties; this provides a direction for molecular photoswitches to achieve higher utilization of the solar spectrum.

## Data Availability

Data are contained within the article or Supplementary Materials.

## Supplementary Materials

The following supporting information can be downloaded at: https://www.mdpi.com/article/10.3390/molecules27103296/s1, Figures S1–S5: Synthetic route to four azobenzene chromophores; Figures S6–S17: NMR spectrum of four azobenzene chromophores; Figure S18: Photographs of the four compounds in two isomeric states; Figures S19–S21: UV–vis absorption spectra of four azobenzene chromophores; Figure S22: First-order kinetic constants for three processes; Figure S23: Charging and discharging cycle diagrams of four azobenzene chromophores; Table S1: Parameters related to the kinetics of partial isomerization.; Videos S1–S4: IR thermal video for low-temperature (−1.0 °C) heat output of four samples.
