# Supplementary material for: Visible Light-Driven Alkyne-Grafted Ethylene-Bridged Azobenzene Chromophores for Photothermal Utilization
Source: Molecules. 2022 May 20;27(10):3296. doi: 10.3390/molecules27103296 (PMC9145641; doi:10.3390/molecules27103296)
Supplement: Supplementary file 1 [file molecules-27-03296-s001.zip › molecules-1728645-supplementary.pdf]

molecules

MDPI

Supplementary Materials

Visible Light-Driven Alkyne-Grafted Ethylene-Bridged Azobenzene Chromophores for Photothermal Utilization

Wenyu Fang, Yiyu Feng *, Jian Gao, Hui Wang, Jing Ge, Qingbin Yang and Wei Feng *

School of Materials Science and Engineering, Tianjin University, Tianjin 300350, China; fangwenyu@tju.edu.cn (W.F.); gj1996@tju.edu.cn (J.G.); huiwang1928@tju.edu.cn (H.W.); gejing_0520@tju.edu.cn (J.G.); yqb514@tju.edu.cn (Q.Y.)

* Correspondence: fengyiyu@tju.edu.cn (Y.F.); weifeng@tju.edu.cn (W.F.)

Experimental

Materials: Unless otherwise noted, the reagents were obtained from commercial sources and used as received. All other chemicals, including 2,2'-ethylenedianiline, were purchased from Bide Pharmatech Ltd., Shanghai, China.

Measurements: The chemical structures of the Azo compounds were characterized using Fourier transform infrared spectroscopy (Tensor 27 spectrometer, Bruker , Billerica, Commonwealth of Massachusetts, USA) in a KBr disc. ^{1}H NMR spectra were collected using a 400 MHz spectrometer (INOVA, Varian , Palo Alto, State of California, USA) with tetramethylsilane as an internal standard. ^{13}C NMR spectra were collected using a 600 MHz spectrome-ter (JNM-ECZ600R/S1, JEOL, Akishima, Japan). HRMS were measured using Thermo Scientific Q Exactive Modular Orbitrap mass Spectrometer (Thermo Fisher Scientific, Waltham, Commonwealth of Massachusetts, USA). The UV–vis ab-sorption spectra of the materials were measured (1 cm path length quartz cuvettes, EA; 330 UV-vis-NIR spectrophotometer, Hitachi , Tokyo, Japan). Differential scan-ning calorimetry (TA Q20, TA instruments , New Castle, State of Delaware, USA) was used to measure the heat flow of the energetically charged *E* isomers of the compounds.

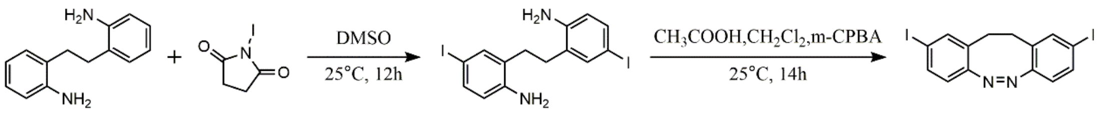

Figure S1. Synthetic route to compounds **1** and **2**.

Synthesis of the intermediate (1): 2,2′-diaminobibenzyl (3 g, 14.1 mmol) was dissolved in DMSO (20 mL) with magnetic stirring at room temperature. To this solution, a solution of N-iodosuccinimide (6.75 g, 30 mmol) was added in DMSO (45 mL) in three portions over 10 min. The mixture was then stirred at room temperature overnight. Next, dichloromethane (20 mL) and deionized water (150 mL) were added and the solution was stirred for 30 min. The resulting solid was separated by vacuum filtration. Silica gel column chromatography was used to purify the solid residue (2:1 ethyl acetate/petroleum ether) and the solvent was removed under reduced pressure to obtain relatively pure compound **1** (5.5 g, 84.4%).

Synthesis of the intermediate (2): 2,2'-(ethane-1,2-diyl)bis(4-iodoaniline) (**1**, 1 g, 2.2 mmol) was added to a mixture of acetic acid (15 mL) and dichloromethane (45 mL) at room temperature, with stirring. meta-Chloroperoxybenzoic acid (m-CPBA, 85% purity, 7.3 g, 36 mmol) was placed into a brown sample bottle, and glacial acetic acid (60 mL) was added to fully dissolve it. Using a syringe pump, this m-CPBA solution (7.3ml, 4.4mmol), was then slowly added to the reaction over 12 h. After the addition was complete, stirring was continued for 2 h. The solvent was removed under reduced

pressure, and the crude product was purified by silica gel column chromatography (1:19 ethyl acetate/petroleum ether). The solvent was removed under reduced pressure to obtain compound **2** as a yellow powder (633 mg, 62.5%) [1].

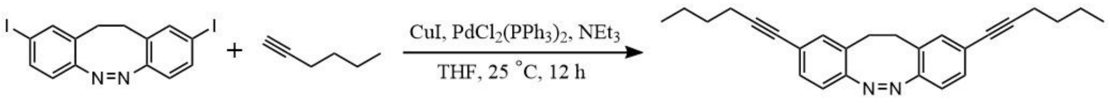

Figure S2. Synthetic route to b-Azo-Q6.

Synthesis of b-Azo-Q6: This step uses the general procedure of the Sonogashira coupling reaction. Compound **2** (460 mg, 1 mmol) was added to ultra-dry tetrahydrofuran (10 mL) and triethylamine (556 µl, 4 mmol) in a three-necked flask. To this, a slight excess of 1-hexyne (234 µl, 2.1 mmol), cuprous iodide (9.5 mg, 0.05 mmol) and bis (triphenylphosphine) palladium(II) dichloride (35.1 mg, 0.05 mmol) were added. The mixture was stirred overnight under an inert gas atmosphere. After the reaction was complete, the solvent was removed under reduced pressure and the residue was purified by silica gel column chromatography (7:93 ethyl acetate/petroleum ether). The solvent was removed under reduced pressure to obtain b-Azo-Q6 as a viscous yellow liquid (313 mg, 85.1%).

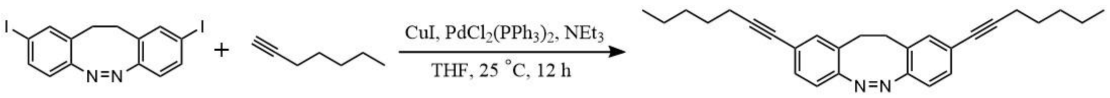

Figure S3. Synthetic route to b-Azo-Q7.

Synthesis of b-Azo-Q7: This step is the same as the synthesis of b-Azo-Q6, but with 1-heptyne (269 µl, 2.1 mmol) instead of 1-hexyne. b-Azo-Q7 was obtained as a viscous yellow liquid (320mg, 80.8%).

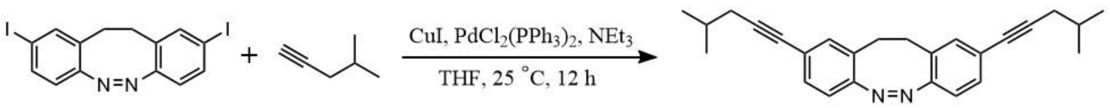

Figure S4. Synthetic route to b-Azo-S6.

Synthesis of b-Azo-S6: This step is the same as the synthesis of b-Azo-Q6, but with 4-methyl-1-pentyne (247 µl, 2.1 mmol) instead of 1-hexyne. b-Azo-S6 was obtained as a viscous yellow liquid (300mg, 81.5%).

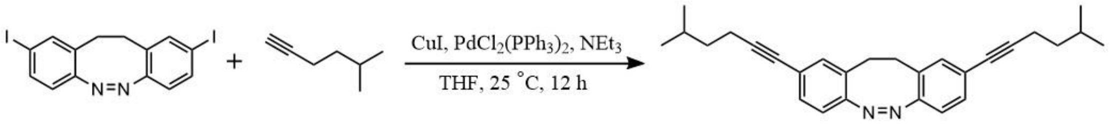

Figure S5. Synthetic route to b-Azo-S7.

Synthesis of b-Azo-S7: This step is the same as the synthesis of b-Azo-Q6, but with 5-methyl-1-hexyne (269 µl, 2.1 mmol) instead of 1-hexyne. b-Azo-S7 was obtained as a viscous yellow liquid (317mg, 80.1%).

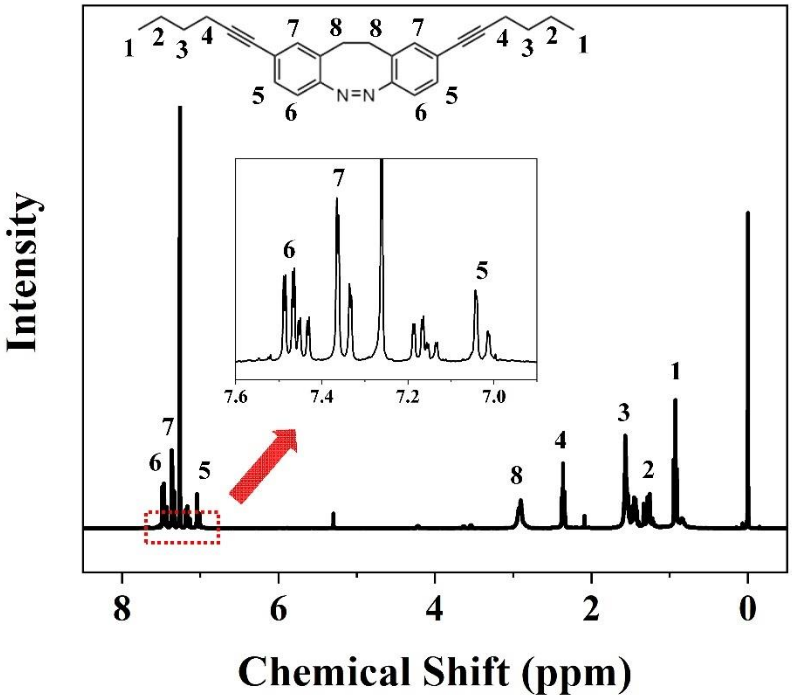

Figure S6. ^{1}H NMR spectrum of b-Azo-Q6.

b-Azo-Q6 ^{1}H NMR (400 MHz, $CDCl_3$, δ): 7.51–7.45 (m, 1H), 7.45–7.41 (m, 1H), 7.03 (dd, J = 11.5, 1.7 Hz, 1H), 2.91 (d, J = 9.6 Hz, 2H), 2.36 (td, J = 7.0, 3.5 Hz, 2H), 1.59–1.52 (m, 2H), 1.45–1.38 (m, 2H), and 0.93 (td, J = 7.3, 1.1 Hz, 3H).

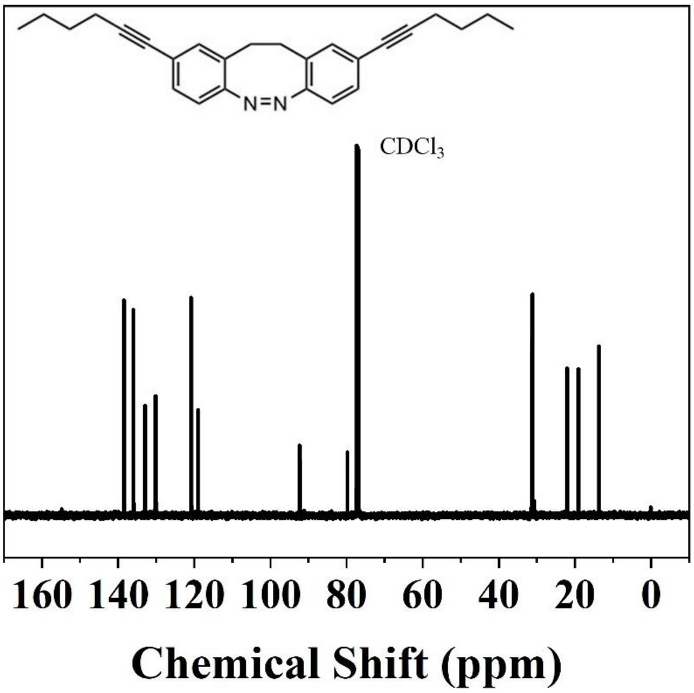

Figure S7. ^{13}C NMR spectrum of b-Azo-Q6.

b-Azo-Q6 ^{13}C NMR (151 MHz, $CDCl_3$, δ): 138.47, 136.00, 132.88, 130.20, 120.81, 119.02, 92.30, 79.78, 31.30, 31.20, 22.08, 19.11, and 13.70.

b-Azo-Q6 HRMS (APCI): $C_{26}H_{29}N_2$ [M+H] calc. 369.2325, found 369.2307.

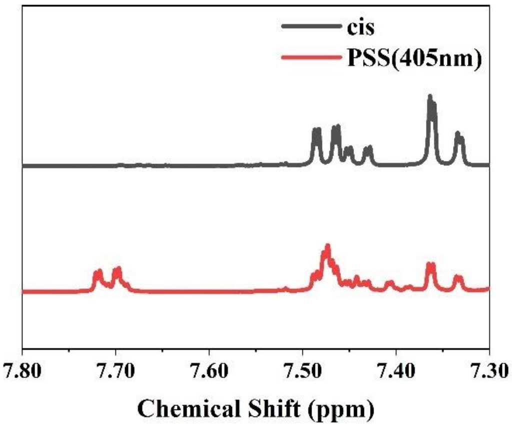

Figure S8. Comparative ¹H NMR spectra of b-Azo-Q6 before and after blue light irradiation.

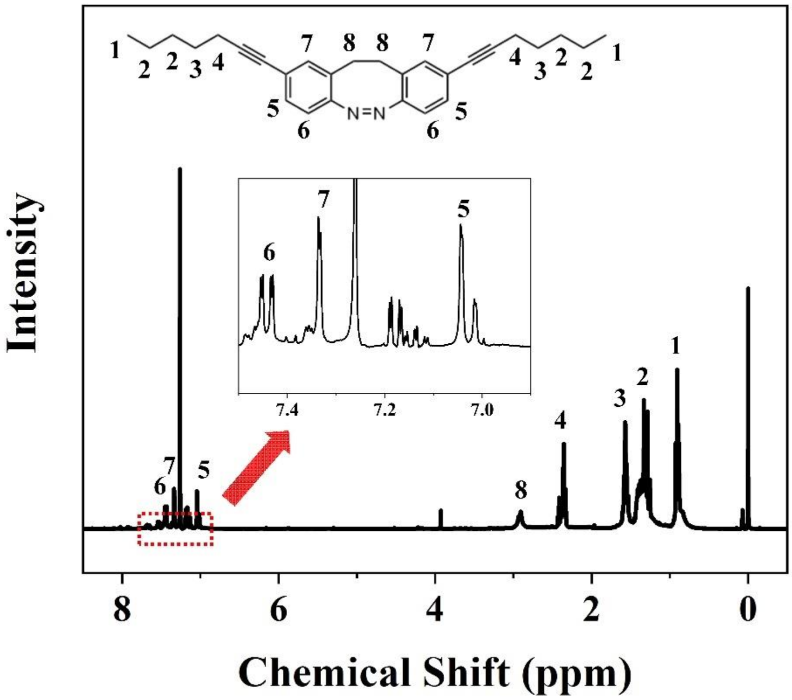

Figure S9. ¹H NMR spectrum of b-Azo-Q7.

b-Azo-Q7 ¹H NMR (400 MHz, $CDCl_3$, δ): 7.57–7.41 (m, 1H), 7.41–7.31 (m, 1H), 7.03 (dd, J = 11.2, 1.7 Hz, 1H), 2.92 (d, J = 10.4 Hz, 2H), 2.46–2.30 (m, 2 H), 1.59–1.49 (m, 2H), 1.47–1.23 (m, 4H), and 0.90 (ddd, J = 9.4, 5.4, 2.6 Hz, 3H).

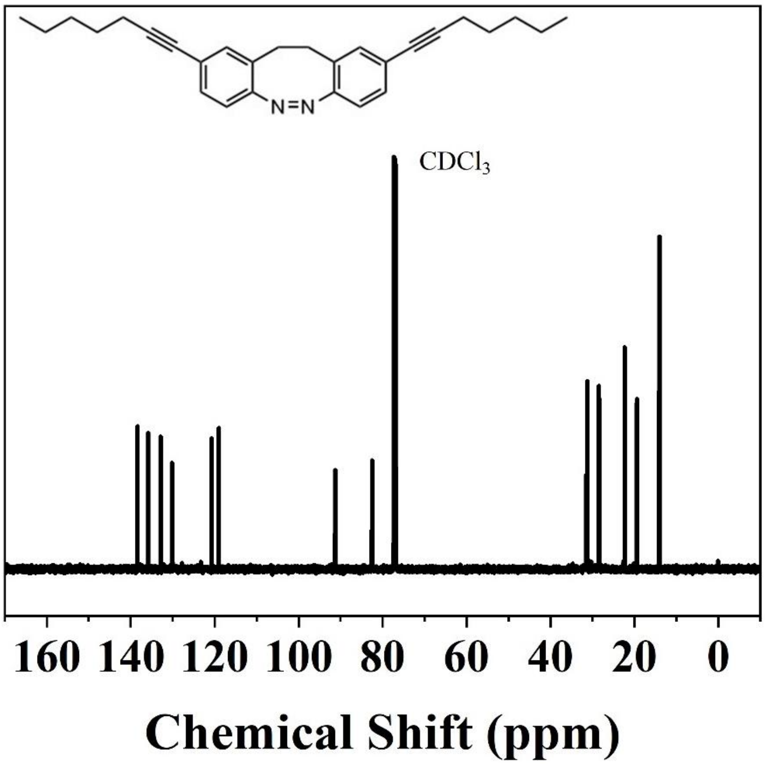

Figure S10. ^{13}C NMR spectrum of b-Azo-Q7.

b-Azo-Q7 ^{13}C NMR (151 MHz, $CDCl_3$, δ): 138.41, 135.81, 132.87, 130.21, 120.77, 119.02, 91.29, 82.51, 31.52, 31.18, 28.44, 22.29, 19.40, and 14.06.

b-Azo-Q7 HRMS (APCI): $C_{28}H_{33}N_2$ [M+H] calc. 397.2638, found 397.2617.

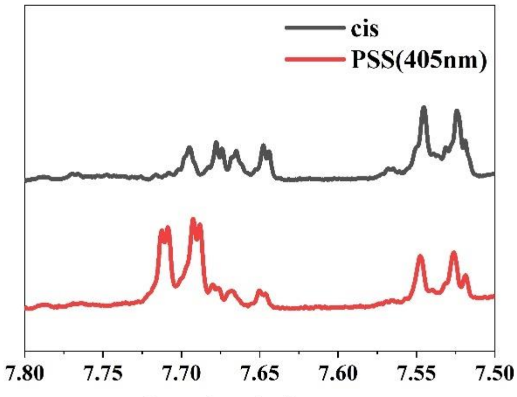

Figure S11. Comparative ^1H NMR spectra of b-Azo-Q7 before and after blue light irradiation.

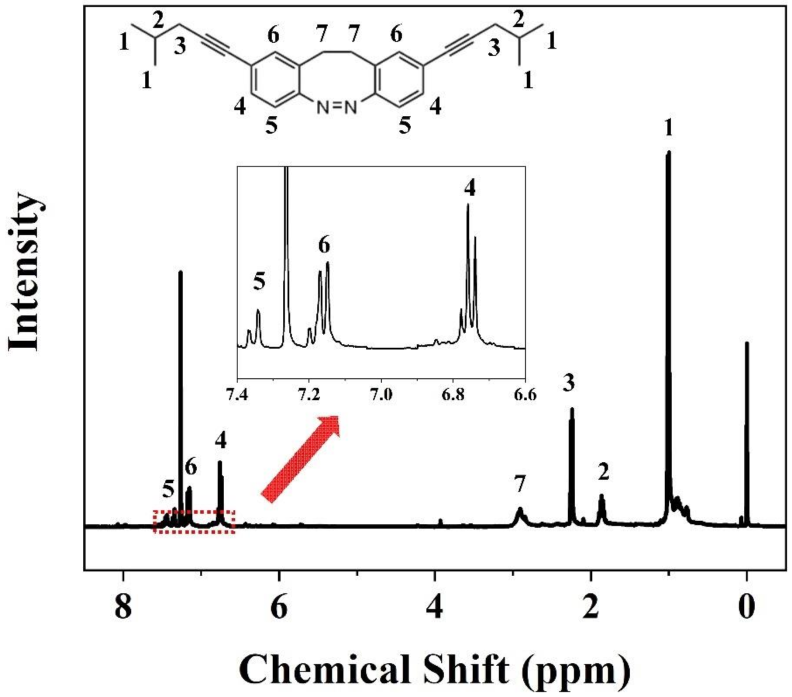

Figure S12. ^{1}H NMR spectrum of b-Azo-S6.

b-Azo-S6 ^{1}H NMR (400 MHz, $CDCl_3$, δ): 7.46 (ddd, J = 12.6, 8.3, 1.8 Hz, 1H), 7.17 (ddd, J = 11.7, 8.1, 1.6 Hz, 1H), 6.76 (t, J = 7.8 Hz, 1H), 2.91 (s, 2H), 2.25 (dd, J = 6.6, 3.6 Hz, 2H), 1.93–1.79 (m, 1H), and 1.01–0.89 (m, 3H).

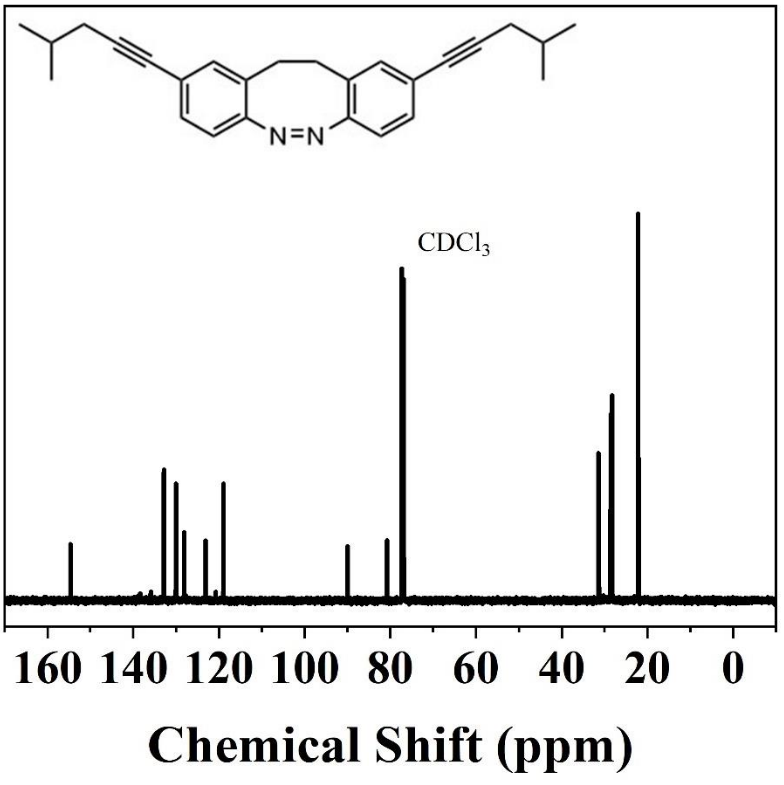

Figure S13. ^{13}C NMR spectrum of b-Azo-S6.

b-Azo-S6 ^{13}C NMR (151 MHz, $CDCl_3$, δ): 154.63, 132.83, 130.03, 128.12, 123.10, 118.96, 89.93, 80.78, 31.41, 28.59, 28.22, and 22.13.

b-Azo-S6 HRMS (APCI): $C_{26}H_{29}N_2$ [M+H] calc. 369.2325, found 369.2311.

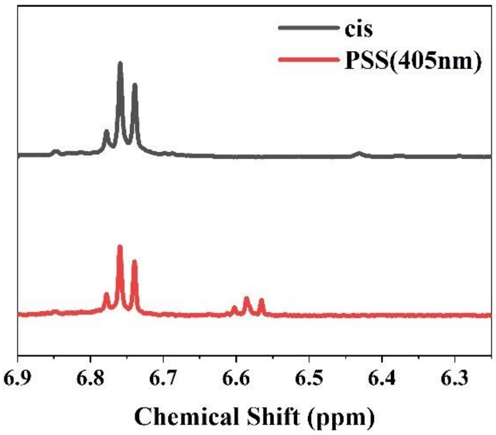

Figure S14. Comparative ^{1}H NMR spectra of b-Azo-S6 before and after blue light irradiation.

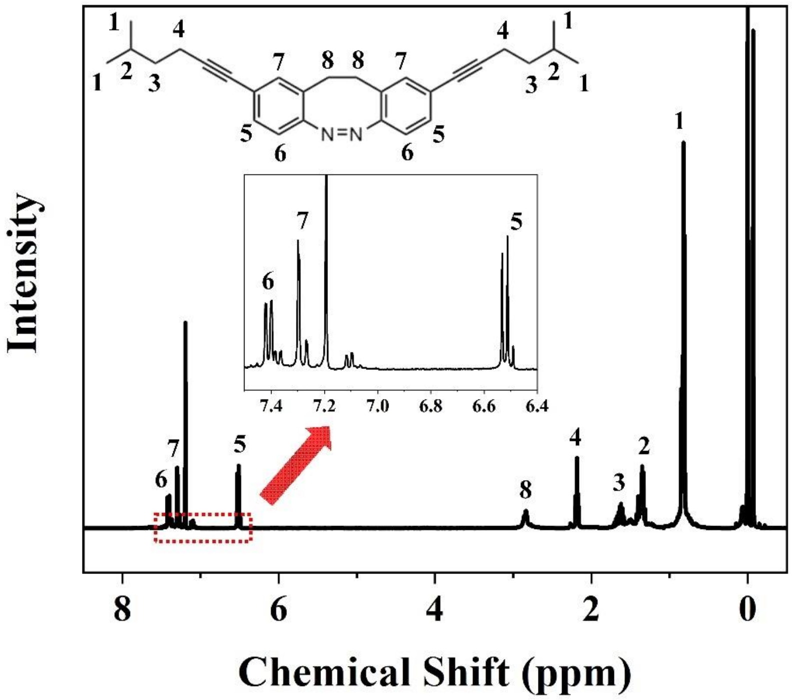

Figure S15. ^{1}H NMR spectrum of b-Azo-S7.

b-Azo-S7 ^{1}H NMR (400 MHz, $CDCl_3$, δ): 7.39 (ddd, J = 13.9, 8.3, 1.8 Hz, 1H), 7.30 (d, J = 1.8 Hz, 1H), 6.51 (t, J = 8.3 Hz, 1H), 2.85 (d, J = 9.4 Hz, 2H), 2.18 (t, J = 7.3 Hz, 2H), 1.64 (ddp, J = 20.2, 13.5, 6.8 Hz, 2H), 1.37 (dq, J = 19.6, 7.2 Hz, 1H), and 0.83 (dd, J = 11.1, 6.7 Hz, 3H).

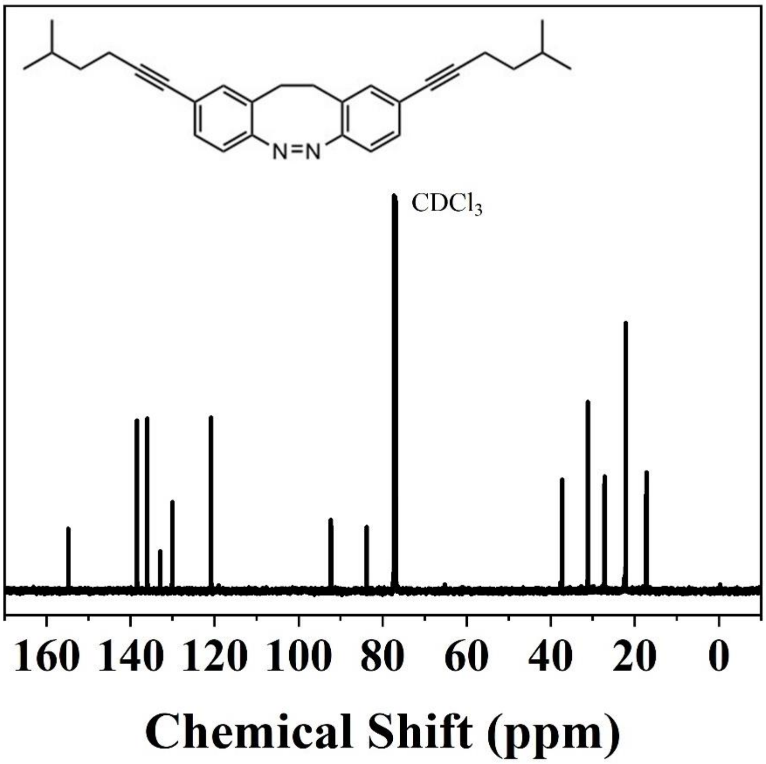

Figure S16. ^{13}C NMR spectrum of b-Azo-S7.

b-Azo-S7 ^{13}C NMR (151 MHz, $CDCl_3$, δ): 154.81, 138.49, 136.01, 132.85, 129.98, 120.81, 92.29, 83.80, 37.24, 31.20, 27.20, 22.17, and 17.26.

b-Azo-S7 HRMS (APCI): $C_{28}H_{33}N_2$ [M+H] calc. 397.2638, found 397.2620.

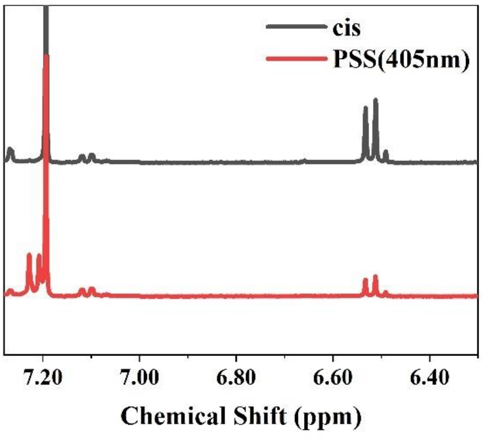

Figure S17. Comparative ^{1}H NMR spectra of b-Azo-S7 before and after blue light irradiation.

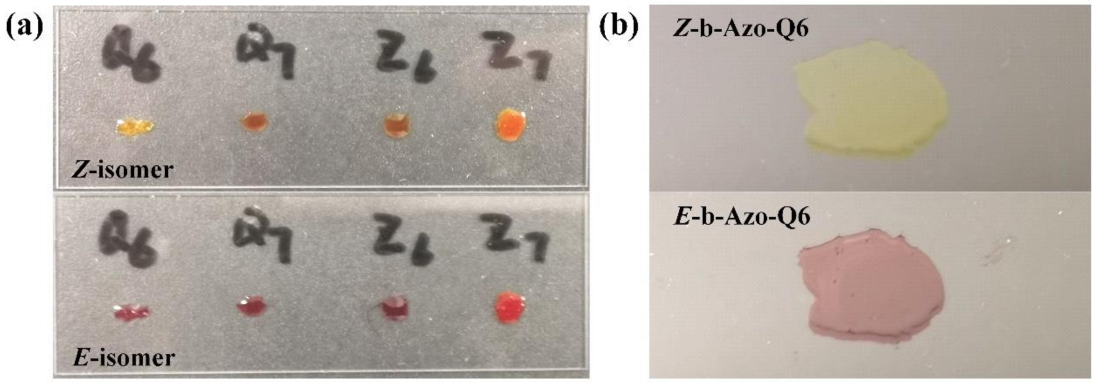

Figure S18. Photographs of the four compounds in two isomeric states.

Characterization of photoisomerization behavior of b-Azo: The b-Azo compound was irradiated with a 500 W point light source (Shenzhen Height-led Optoelectronics Technology Co., Ltd., Shenzhen, China); 405 and 520 nm lamps were selected. Time intervals were recorded, and a small amount of samples were scraped with toothpicks at specific time points and dissolved in ethyl acetate for testing. The light source was placed 10 cm above the sample. The light intensities were: 405 nm, 24.01 mW/cm^2; 520 nm, 5.06 mW/cm^2. The temperature was 25 °C. A spectrophotometric analysis of the charging process was performed using 405 nm blue light irradiation at room temperature. A spectrophotometric analysis of the heat recovery process under ambient conditions was performed by covering a sample of the *E* isomer with tin foil and keeping it in the dark while the measurements were taken. A spectrophotometric analysis of the heat recovery process was also performed under 520 nm green light irradiation at room temperature. The resultant spectra for the three processes are shown in Figures S19—21. The first-order kinetic constants that were calculated according to Equation (S1, S2) are shown in Figure S22 [2] and some of the relevant parameters can be found in Table S1.

The heat charging process of azobenzene is basically in accordance with the following Equation (S1):

$$ln\left(\frac{A_0-A_t}{A_0-A_\infty}\right) = -k_{rev}t \quad \text{(S1)}$$

Similarly, the law of the reversion process of azobenzene can be expressed by the following Equation (S2):

$$ln\left(\frac{A_t-A_\infty}{A_0-A_\infty}\right) = -k_{rev}t \quad \text{(S2)}$$

Among them, A_0 is the absorbance value after complete heat charging by blue light irradiation in the state without solvent assistance, A_t is the absorbance value at time t, and A_∞ is the absorbance value of azobenzene in a completely stable state before blue light irradiation.

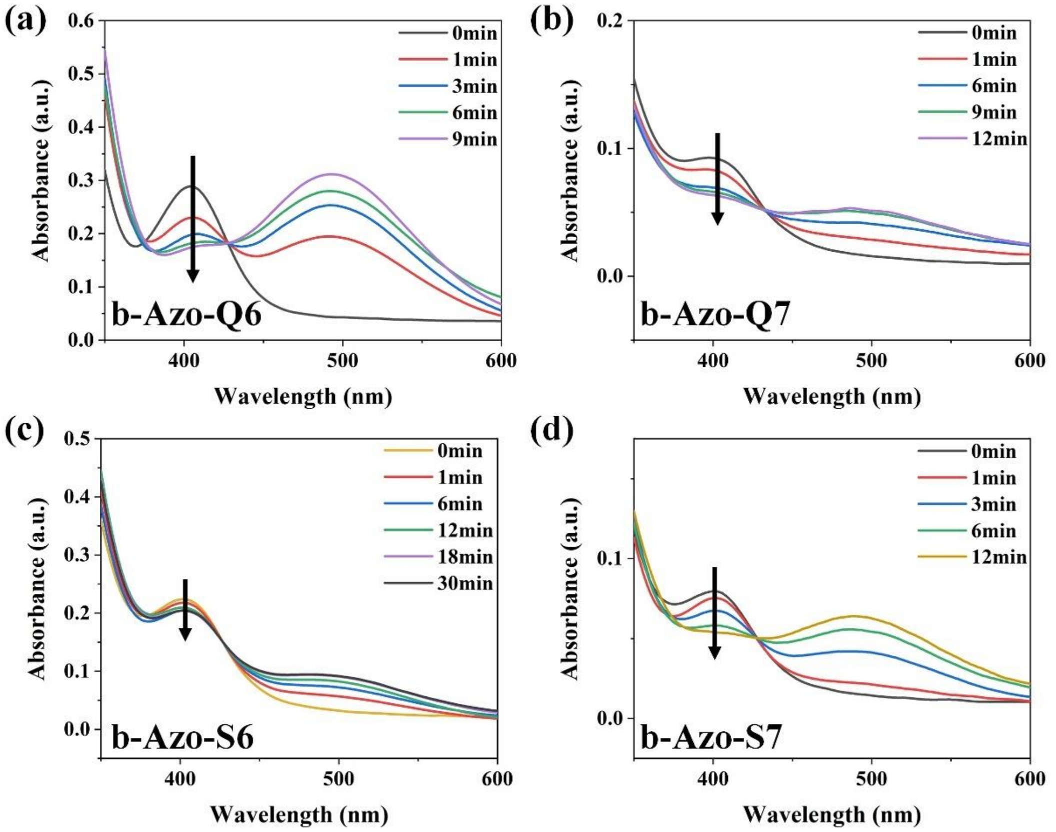

Figure S19. UV–vis absorption spectra of the charging process under blue light irradiation.

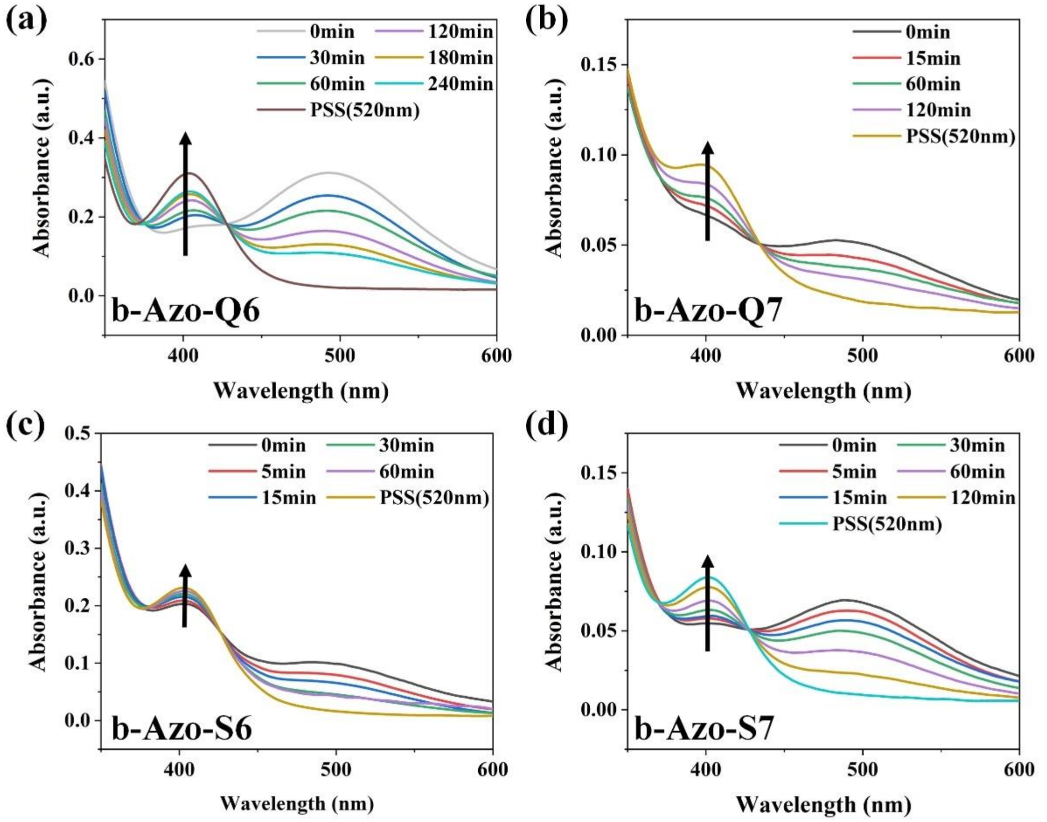

Figure S20. UV–vis absorption spectra of the recovery process under ambient conditions.

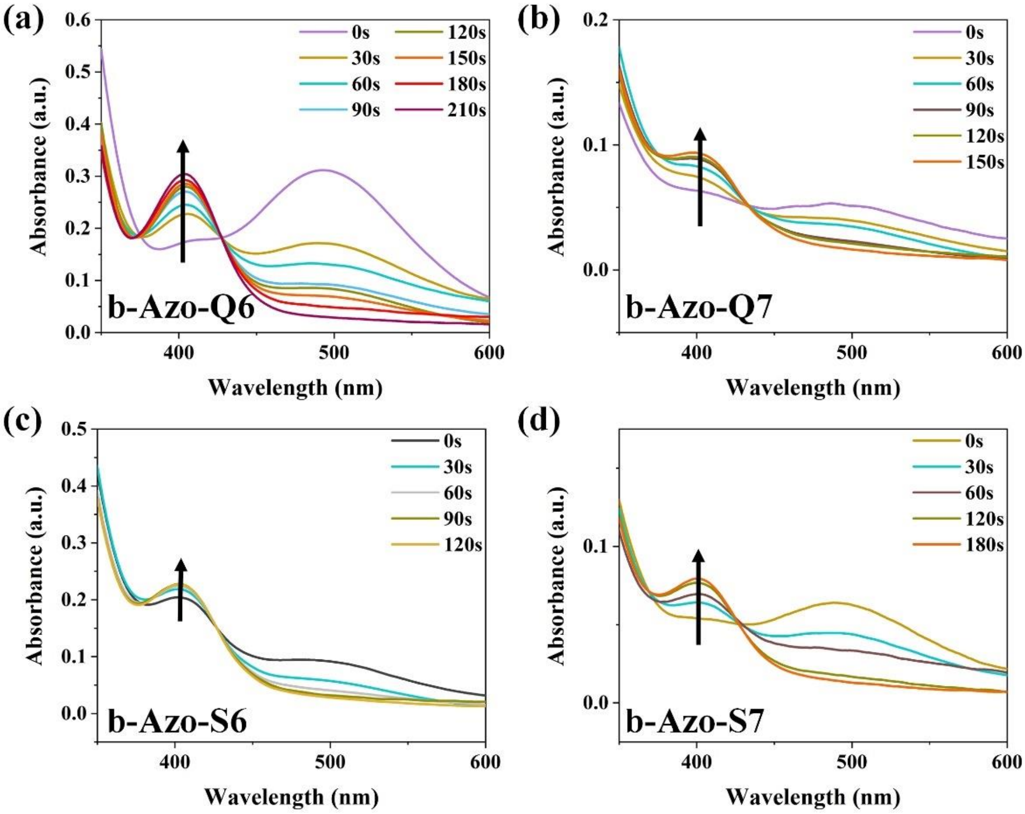

Figure S21. UV–vis absorption spectra of the recovery process under green light irradiation.

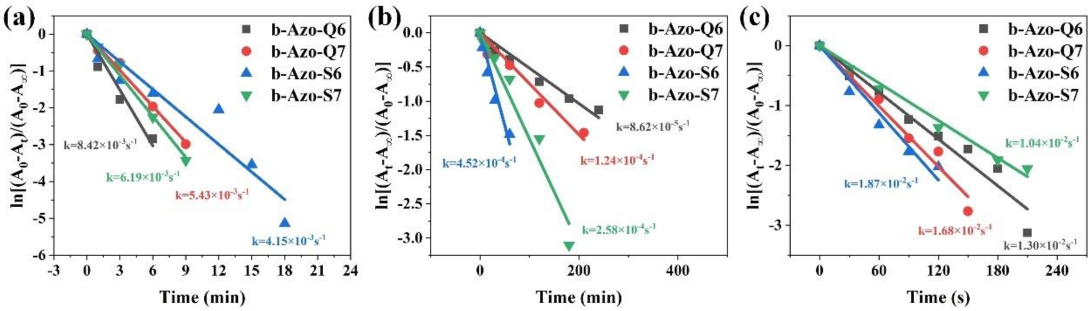

Figure S22. First-order kinetic constants for three processes: (a) charging process under blue light irradiation, (b) heat recovery processes under ambient conditions, and (c) heat recovery processes under green light irradiation.

Table S1. Parameters related to the kinetics of partial isomerization.

Material	405 nm-$t_{1/2}$ (min)	$t_{1/2}$ (min)	520 nm-$t_{1/2}$ (s)	D_{imax} (%) Solvent free	D_{imax} (%) Solvent assisted
b-Azo-Q6	1.37	133.3	53.3	58.5	59.6
b-Azo-Q7	2.12	91.6	41.2	42.8	42.9
b-Azo-S6	2.78	25.6	37.1	27.4	52.8
b-Azo-S7	1.86	55.2	66.6	56.4	57.1

Cyclic stability of isomerization: From initial UV–vis absorption spectra of the compounds, the intensities of the maximum absorption peaks in the blue and in the green regions were recorded. The compounds were irradiated with blue light for 15 min and the blue and green absorption maxima were recorded. The compounds were then irradiated

with green light for 5 min, and the absorption maxima were recorded again. This cycle of blue and green light irradiation was repeated 10 times to demonstrate the cycle stability of the compounds, as shown in Figure S23.

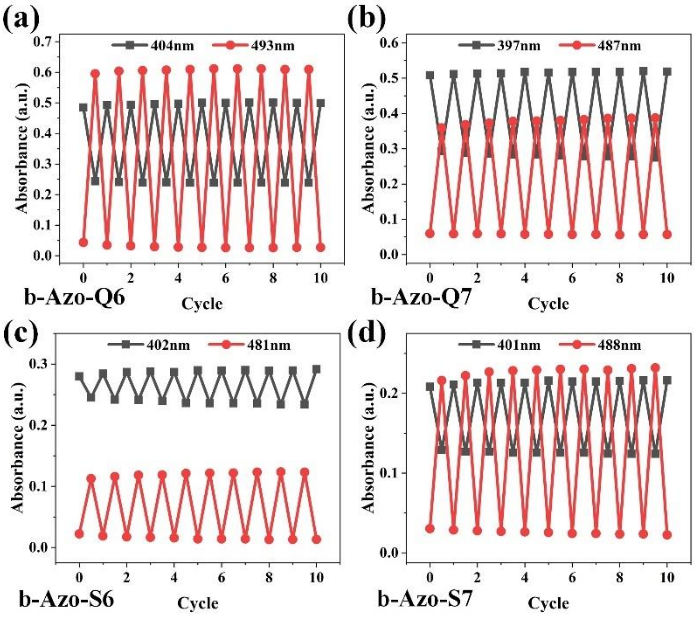

Figure S23. Charging and discharging cycle diagrams of b-Azo-Q6 (a), b-Azo-Q7 (b), b-Azo-S6 (c), and b-Azo-S7 (d).

Visual characterization of infrared thermal imaging camera temperature changes: Real-time temperature changes during the sample exotherm were monitored by infrared thermography for visual characterization. A 20 mg sample was weighed and coated onto a quartz sheet, and the sample was irradiated using a blue point light source (405 nm, 24.01 mW/cm^2) for 15 min. After heat charging, it was placed on a semiconductor-cooled thermostatic cold table set at -3 °C. The sample temperature was allowed to stabilize; at this time, the central temperature of the sample was maintained at approximately -1 °C. The sample was irradiated with a green point light source (520 nm, 5.06 mW/cm^2) to stimulate the heat discharging process, while the temperature change during the sample exotherm was monitored using an infrared thermal imaging camera. The results are shown in Videos S1–4.

Video S1. IR thermal video for low-temperature (-1.0 °C) heat output of *E*-b-Azo-Q6.

Video S2. IR thermal video for low-temperature (-1.0 °C) heat output of *E*-b-Azo-S6.

Video S3. IR thermal video for low-temperature (-1.0 °C) heat output of *Z*-b-Azo-Q6.

Video S4. IR thermal video for low-temperature (-1.0 °C) heat output of *Z*-b-Azo-S6.

Reference

1. Maier, M.S.; Hull, K.; Reynders, M.; Matsuura, B.S.; Leippe, P.; Ko, T.; Schaffer, L.; Trauner, D. Oxidative Approach Enables Efficient Access to Cyclic Azobenzenes. *J. Am. Chem. Soc.* **2019**, *141*, 17295-17304, doi:10.1021/jacs.9b08794.
2. Stuart, C.M.; Frontiera, R.R.; Mathies, R.A. Excited-state structure and dynamics of cis- and trans-azobenzene from resonance Raman intensity analysis. *J. Phys. Chem. A* **2007**, *111*, 12072-12080, doi:10.1021/jp0751460.
